# Contemporary functional neuroanatomy and pathophysiology of dystonia

**DOI:** 10.1007/s00702-021-02299-y

**Published:** 2021-01-24

**Authors:** Norbert Brüggemann

**Affiliations:** 1 Department of Neurology, University of Lübeck, Ratzeburger Allee 160, 23538 Lübeck, Germany; 2 Institute of Neurogenetics, University of Lübeck, Lübeck, Germany; 3 Center for Brain, Behavior and Metabolism, University of Lübeck, Lübeck, Germany

**Keywords:** Dystonia, Pathophysiology, Cerebellum, Basal ganglia, Inhibition

## Abstract

Dystonia is a disabling movement disorder characterized by abnormal postures or patterned and repetitive movements due to co-contraction of muscles in proximity to muscles desired for a certain movement. Important and well-established pathophysiological concepts are the impairment of sensorimotor integration, a loss of inhibitory control on several levels of the central nervous system and changes in synaptic plasticity. These mechanisms collectively contribute to an impairment of the gating function of the basal ganglia which results in an insufficient suppression of noisy activity and an excessive activation of cortical areas. In addition to this traditional view, a plethora of animal, genetic, imaging and electrophysiological studies highlight the role of the (1) cerebellum, (2) the cerebello-thalamic connection and (3) the functional interplay between basal ganglia and the cerebellum in the pathophysiology of dystonia. Another emerging topic is the better understanding of the microarchitecture of the striatum and its implications for dystonia. The striosomes are of particular interest as they likely control the dopamine release via inhibitory striato-nigral projections. Striosomal dysfunction has been implicated in hyperkinetic movement disorders including dystonia. This review will provide a comprehensive overview about the current understanding of the functional neuroanatomy and pathophysiology of dystonia and aims to move the traditional view of a ‘basal ganglia disorder’ to a network perspective with a dynamic interplay between cortex, basal ganglia, thalamus, brainstem and cerebellum.

## Introduction

Dystonia belongs to the group of hyperkinetic movement disorders that is characterized by abnormal postures or patterned and repetitive movements due to sustained or intermittent muscle contractions (Albanese et al. 2013). The central problem in dystonia is the exaggerated activation of muscle groups that are required for the execution of a certain movement. The additional co-contraction of muscles in proximity to the muscles desired for a certain movement and the simultaneous activation of antagonistic muscles reinforce involuntary dystonic movements and postures and contribute to the loss of voluntary control over motor actions. Dystonia does, however, not only appear in the context of intended or goal-directed movements such as in focal hand dystonia or spasmodic dysphonia but may also occur at rest, e.g. in cervical dystonia. Dystonias can affect almost every part of the body. Focal dystonias are restricted to one body region, e.g. blepharospasm, cervical dystonia or focal hand dystonia. Dystonia of a single body region may, however, spread to other body parts and becomes then segmental, multifocal or generalized (Berman et al. 2020). The tendency for spread depends on the involved body region and appears to be highest for the eyes.

In isolated dystonias, previously referred to as primary dystonia, dystonia is the only movement disorder aside from accompanying tremor (Albanese et al. 2013). In contrast, combined dystonias present with additional movement disorders, mostly parkinsonism, myoclonus or chorea. Complex dystonias are a group of heterogeneous conditions where dystonia is only one feature in addition to other clinical manifestations.

The etiology of dystonias is heterogeneous and includes acquired, genetic and sporadic forms. The acquired forms result from injuries of critical neuronal structures, whereas genetic forms are associated with pathogenic mutations in dystonia-causing genes. Discoveries in these two etiologies have largely supported the understanding of the underlying functional neuroanatomy and pathophysiology of dystonias. The most common clinical constellation in dystonia, however, is the absence of neuronal lesions and disease-causing mutations. In these sporadic cases, complex gene–environment interactions are discussed to cause the disease. First genome-wide association studies revealed an association of sporadic dystonias with genetic risk factors (Mok et al. 2014; Lohmann et al. 2014) but the evidence is still limited.

This review will provide a comprehensive overview about the current understanding of the functional neuroanatomy and pathophysiology of dystonia. The article will discuss recent advances in dystonia genetics, evidence from animal models, lesional and histopathological studies in humans, structural and functional neuroimaging and relevant electrophysiological findings. The article focuses on the systems level and aims to move the traditional view of a ‘basal ganglia disorder’ (Krystkowiak et al. 1998) to a network perspective with a dynamic interplay between cortex, basal ganglia, thalamus, brainstem and cerebellum.

## The traditional view: disorganization of the sensorimotor system, impaired inhibition and maladaptive plasticity

Several abnormalities in the sensorimotor system are present in patients with dystonia arguing that an abnormal integration of sensory information is associated with the occurrence of dystonic symptoms. A good example to illustrate changes in sensorimotor integration is the presence of an alleviating maneuver (sensory trick) in the vast majority of patients with dystonia (Patel et al. 2014). Clinically relevant and overt sensory deficits are, however, usually not observed. One major challenge is that such abnormalities can be observed in other brain disorders as well and may thus not be causal but rather a consequence of dystonic muscle activity.

On a behavioral level, patients with dystonia exhibit an abnormal temporal discrimination (Hutchinson et al. 2013) associated with hyperactivity in the basal ganglia (Peller et al. 2006). This abnormality can also be found in a proportion of unaffected relatives which highlights that part of the genetic background is shared and associated with the underlying dystonic trait rather than the state. In keeping with this notion, the somatotopic representation in the sensorimotor cortex of dystonia patients is disorganized with overlapping receptive fields indicating impaired neuronal selectivity (Neychev et al. 2011). Another important concept in dystonia is the loss of inhibition on multiple levels of the central nervous system including cortex, basal ganglia, brainstem and spinal cord (Hallett 2011). Surround inhibition on the cortical and basal ganglia level is required to select an appropriate command to the motor cortex in order to execute a specific and intended movement and thus to implement goal-directed behavior. This ‘funneling’ or gating function of the basal ganglia is impaired in dystonia which results in an insufficient suppression of surrounding noisy activity, an excessive activation of cortical areas and subsequent co-contractions of muscle groups that otherwise should not be active (Sohn and Hallett 2004). The inhibitory deficit in dystonia is probably associated not only with decreased GABA levels in the basal ganglia but also the cerebellum (Levy and Hallett 2002; Gallea et al. 2018) although conflicting data were published (Herath et al. 2010).

A plethora of studies have found maladaptive plasticity in the striato-pallido-thalamo-cortical loop of dystonia patients using different non-invasive brain stimulation techniques (Mink 2018). Recent evidence suggested similar maladaptive changes in the cerebellum (Porcacchia et al. 2019). The intra- and inter-individual variability of plasticity response in both, patients and controls, however, underlines the complexity of this concept and highlights that several individual factors have a considerable influence.

## Investigating carriers of mutations in dystonia-related genes

Several genes were identified to cause monogenic isolated dystonia. Four of these genes, *TOR1A*, *THAP1*, *GNAL* and *ANO3*, were independently replicated and validated (Domingo et al. 2020). The *KMT2B* gene occupies a special position as it is either associated with isolated dystonia or most commonly a more complex phenotype including microcephaly, intellectual and developmental delay and other movement disorders, e.g. chorea or myoclonus (Zech et al. 2019). Other genes still await their independent replication. The investigation of patients with dystonia including manifesting carriers (MCs) of mutations in dystonia-causing genes is strongly hampered by continuous dystonic movements. Likewise, microstructural changes of the white or gray brain matter in MCs could be rather a consequence than a primary event in the pathophysiology of dystonia. Investigations in non-dystonic extremities (Vo et al. 2015) have been conducted to circumvent the presence of continuous muscle activity-related neural activation. The major advantage of the monogenic dystonia forms is, however, that non-manifesting mutation carriers (NMCs) who are free of any confounding movement disorder can be studied as well.

In this context, the metabolic activity is increased in the striatum and cerebellum of *TOR1A* NMCs, whereas the metabolism is decreased in the putamen, thalamus, upper brain stem and cerebellum of *THAP1* NMCs (Carbon and Eidelberg 2009; Carbon et al. 2004b). Interestingly, the reduction of putaminal activity was even more pronounced in manifesting *THAP1* MCs (Carbon et al. 2004b). On the cortical level, an increased activity was present in the pre-SMA and parietal cortex of *TOR1A* and *THAP1* MCs compared to NMCs indicating that cortical activity changes are associated with penetrance of the underlying mutation (Carbon et al. 2004b). Using a principal component analysis approach to identify a specific dystonia-related metabolic pattern (DYT-RP) revealed, not surprisingly, an increased DYT-RP expression in *TOR1A* and *THAP1* MCs and a reduced expression in NMCs even when compared to controls. This highlights that adaptive changes occur in dystonia-related sensorimotor brain regions that prevent NMCs from manifesting the disease.

Diffusion tensor imaging and tractography demonstrated an abnormal structural integrity of the white matter not only in patients with *TOR1A (*Carbon et al. 2004a; Argyelan et al. 2009) and *THAP1*-related (Argyelan et al. 2009; Carbon et al. 2004a) dystonia but also in symptomatic carriers of variants in the yet unconfirmed *COL6A3* gene (Jochim et al. 2018). Changes were observed in the subgyral white matter of the sensorimotor cortex (Carbon et al. 2004a) and the dorsal tegmentum of the pons adjacent to the superior cerebellar peduncle as well as in the ponto-cerebellar tract (Sako et al. 2015). One of the most intriguing findings was, however, a reduced integrity of the cerebello-thalamo-cortical fiber tracts which was present in *TOR1A* mutation carriers regardless of whether they were manifesting or not although the changes were more pronounced in MCs. These microstructural alterations were associated with increased motor activation responses arguing for a loss of inhibition on a cortical level due to a cerebellar dis- or hypo-connection (Argyelan et al. 2009). These findings are well in line with a *dyt1* knock-in mutant mouse model where similar structural changes could be observed in the cerebello-thalamic, thalamo-cortical and thalamo-striatal tract in the absence of a motor phenotype (Ulug et al. 2011). The cerebellar disconnection hypothesis is corroborated by an increased metabolic activity in the cerebellum of MCs, NMCs and the *dyt1* knock-in mutant mouse (Eidelberg et al. 1998; Ulug et al. 2011; Argyelan et al. 2009; Carbon et al. 2008; Odorfer et al. 2019; Sako et al. 2015) that is inversely correlated with the integrity of the cerebello-thalamic connection (Argyelan et al. 2009; Ulug et al. 2011). Conflicting findings were reported in myoclonus-dystonia due to *SGCE* mutations along with increased white matter volume in the area between the cerebellum and the thalamus (van der Meer et al. 2012). Further evidence for a crucial role of the cerebello-thalamic projection in the pathophysiology of dystonia comes from X-linked dystonia-parkinsonism (XDP), an inherited adult-onset neurodegenerative disorder with striking striatal and pallidal volume loss (Hanssen et al. 2018, 2019) and widespread pathology of the white matter (Bruggemann et al. 2016; Blood et al. 2018) due to a mutation in the *TAF1* gene (Aneichyk et al. 2018; Westenberger et al. 2019). Imaging studies revealed additional atrophy in the associative part of cerebellum, whereas hypertrophic changes were observed in the sensorimotor part and the dorsal pontine tegmentum (Hanssen et al. 2018). In contrast to non-degenerative DYT-Tor1A, increased gray matter volumes probably indicate a compensatory mechanism that may counteract the pathological striato-pallido-thalamo-cortical circuit in XDP.

## The microstructure of the striatum and its implications in the pathophysiology of dystonia

**Fig. 1:**
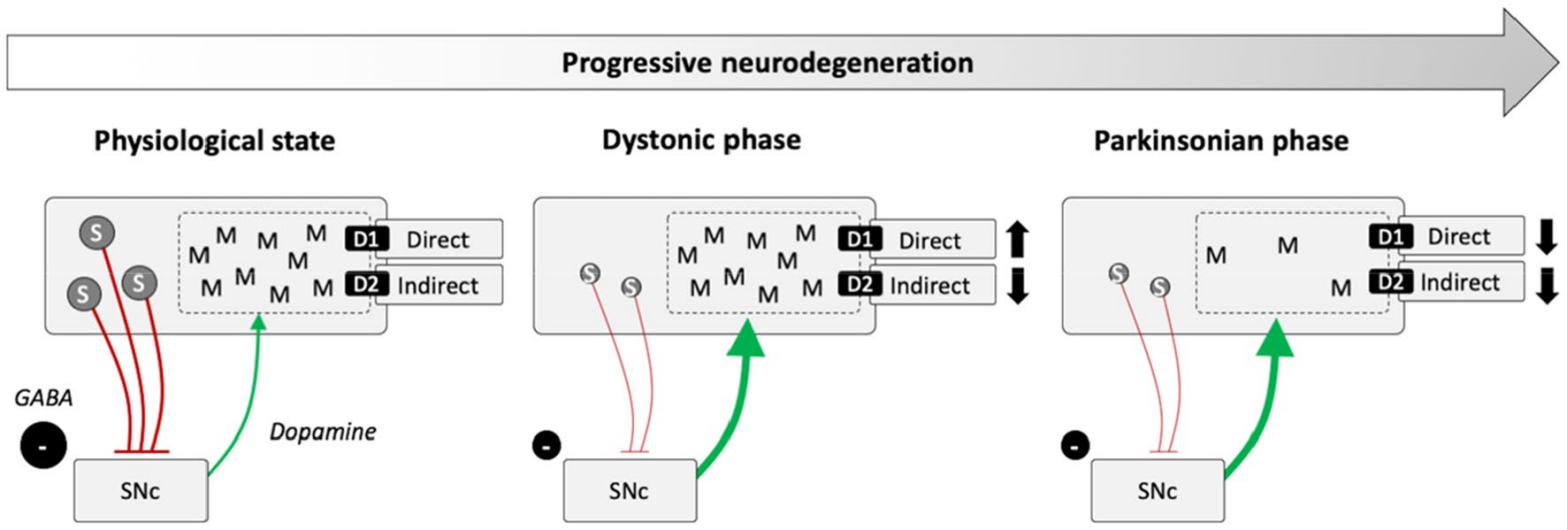
Proposed functional alterations in X-linked dystonia parkinsonism over the disease course due to progressive striatal neurodegeneration. In the physiological state, neurons of the striosomes (S) send inhibitory GABAergic projections to dopamine-containing neurons of the SNc. The SNc innervates D1 and D2 receptor containing matrix (M) neurons and thus differentially modulates the balance between the direct and indirect pathway. Predominant degeneration of the striosomes results in a postulated SNc disinhibition in the early phase of XDP. Dopaminergic dysregulation shifts the balance between both pathways towards the direct pathway which results in impaired surround inhibition and facilitates dystonia. The degeneration of matrix neurons in later disease stages leads to MSA-like parkinsonism due to reduced postsynaptic dopamine receptor density. *S* striosomes, *M* matrix, *SNc* substantia nigra pars compacta, *D1* dopamine D1 receptor, *D2* dopamine D2 receptor, *GABA* gamma aminobutyric acid, *MSA* multiple system atrophy

The striatal microstructure comprises two neurochemically defined compartments, the striosome and the matrix. Histopathological studies across species including mice, rats, monkeys and humans consistently demonstrate that the striosome occupies 10–15% of the entire striatal volume (Johnston et al. 1990; Crittenden and Graybiel 2011; Desban et al. 1993). Striosome and matrix are distributed across the striatum in a patchy fashion; however, the density of striosomes appears to follow a caudo-rostral gradient with the rostral striatum being considered striosome-rich and the dorsolateral putamen mainly comprising matrix (Johnston et al. 1990; Desban et al. 1993). However, data on the distribution of striosomes in the dorsolateral putamen in humans is lacking (Holt et al. 1997). Striosomal neurons receive synaptic input from the prelimbic cortex and are the origin of GABAergic striatonigral projections that form bouquet-like arborizations with dopamine-containing neurons in the substantia nigra pars compacta (SNc) (McGregor et al. 2019; Crittenden et al. 2016; Matsushima and Graybiel 2020). This suggests that the dopaminergic tone in the SNc is controlled by this striosome-derived striatonigral pathway. Dopaminergic neurons in turn project to the striatum via the nigrostriatal pathway with most of the fibers targeting the dorsolateral putamen. As a consequence, impairment of inhibitory striatonigral projections due to striosomal dysfunction may result in nigral disinhibition and presumed overactivity of dopaminergic nigrostriatal projections. Dopamine excess in the dorsolateral putamen with a predominance of matrix neurons could give rise to an imbalance of the direct and indirect pathway (Fig. 1). Matrix neurons express dopamine D1 and D2 receptors, innervate the globus pallidus externus, internus and the SN pars reticulata and are thus directly rendering the direct (excitatory) and indirect (inhibitory) cortico-subcortical pathways (Lanciego et al. 2012). Accordingly, dopaminergic dysregulation has been associated with dystonia in a *dyt1* dystonia animal model (Ip et al. 2016). Striosomal dysfunction has also been implicated in early stages of Huntington’s disease (Hedreen and Folstein 1995), and imbalance between striosomal and matrix function are discussed as potential disease mechanisms in human dystonia and Levodopa-induced dyskinesia in patients with advanced Parkinson’s disease (Henry et al. 2003; Sato et al. 2008). Moreover, post-mortem investigations of brains of deceased XDP patients revealed extensive striatal atrophy with a consistent loss of striosomes in the early phase of XDP, whereas degeneration of the matrix is additionally present in later disease stages (Goto et al. 2005) (Fig. 1). This is in line with gradual putaminal neurodegeneration including severe and more advanced neurodegeneration in the anteromedial, striosome-enriched part compared to the matrix-enriched dorsolateral putamen during the dystonic phase of the disease (Hanssen et al. 2019). In Dopa-responsive dystonia (DRD, Segawa syndrome), an inherited neurometabolic disorder with a clinical presentation of combined dystonia-parkinsonism, mutations in different genes of the pteridine and catecholamine pathway result in striatal dopamine deficiency and dystonia. In a DRD mouse model, major depletion of tyrosine hydroxylase (TH) was present in the striatum along with a dystonic phenotype (Sato et al. 2008). In regions with residual TH labeling the TH loss was more pronounced in the striosomes compared to matrix linking striosomal pathology and dopamine depletion to deficient GABAergic inhibition of the striatonigral pathway with a subsequent shift towards the direct pathway in the matrix where TH activity was relatively preserved. Further research is, however, warranted to clarify the role of striosome vs. matrix dysfunction in dystonia as an experimental blockade of striosomal projection neurons did not elicit involuntary hyperkinetic movements in rodents (Xiao et al. 2020) although locomotor performance was more variable and habitual behaviors were disrupted (Nadel et al. 2020).

## The role of the cerebellum

Strong evidence for a causal role of the cerebellum in the pathophysiology of dystonia results from animal models (Bologna and Berardelli 2018). Several manipulations have been conducted to investigate the contribution of the cerebellum to dystonia. Microinjections of the glutamate agonist kainic acid and the application of sodium pump blockers in the cerebellum induced dystonia-like behaviors in rodents (Alvarez-Fischer et al. 2012; Calderon et al. 2011; Neychev et al. 2008). In keeping, RNA-mediated knockdown of the α3-containing sodium pumps led to a phenotype of dystonia-parkinsonism (Fremont et al. 2015) which corresponds to the human disease of rapid-onset dystonia-parkinsonism caused by mutations in the gene encoding the α3-subunit of the sodium–potassium pump (Brashear et al. 2007). Dystonia could also be provoked in a number of genetic rodent models including the *dt* mouse (LeDoux et al. 1998; Oltmans et al. 1984) and the tottering mouse harboring a mutation in the *CACNA1A* gene (Neychev et al. 2008). On the other hand, structural cerebellar abnormalities were identified in *Dyt1* ΔGAG knock-in mice (Zhang et al. 2011; Song et al. 2014; Vanni et al. 2015) as well as in a Purkinje cell-specific *Dyt1* knockout (Zhang et al. 2011; Vanni et al. 2015) and *THAP1* knock-in (Ruiz et al. 2015) as well as knock-out mouse models (Frederick et al. 2019).

Several reports of patients with cerebellar lesions due to ischemic or hemorrhagic stroke (O’Rourke et al. 2006; Waln and LeDoux 2010; Bana et al. 2015) or a cerebellar mass (Alarcon et al. 2001) have long been associated with dystonia. Lesion network mapping in 25 patients with secondary cervical dystonia found that dystonia-associated lesions had a heterogeneous localization including cerebellum, brainstem and basal ganglia. They were, however, all part of a single functionally connected brain network with positive connectivity to the cerebellum and negative connectivity to the somatosensory cortex (Corp et al. 2019).

Dystonia is also a frequent clinical feature in neurodegenerative diseases of the cerebellum. Among the autosomal dominant spinocerebellar ataxias (SCAs), dystonic signs can most frequently be observed in SCA type 3 (Kuo et al. 2017; Rossi et al. 2014). In late-onset forms of autosomal recessive ataxia teleangiectasia, dystonia can be the presenting clinical manifestation even in the absence of ataxia and teleangiectasias thus mimicking isolated generalized dystonia (Necpal et al. 2018; Saunders-Pullman et al. 2012; Meissner et al. 2013). Over the disease course, dystonia occurs in almost 90% of patients with ataxia teleangiectasia but also chorea as another hyperkinetic movement disorder can be frequently observed (Levy and Lang 2018). The response of dystonia to levodopa in single cases with ataxia teleangiectasia suggests an involvement of the cerebellar dopaminergic system in the etiology of dystonia (Charlesworth et al. 2013; Thompson et al. 2014).

## Cerebellum–basal ganglia crosstalk

The traditional view on neural network organization encompasses distinct striato-pallido-thalamo-cortical and cerebello-thalamo-cortical pathways that convergently project to distinct thalamic nuclei and are only integrated at the neocortical level.

**Fig. 2:**
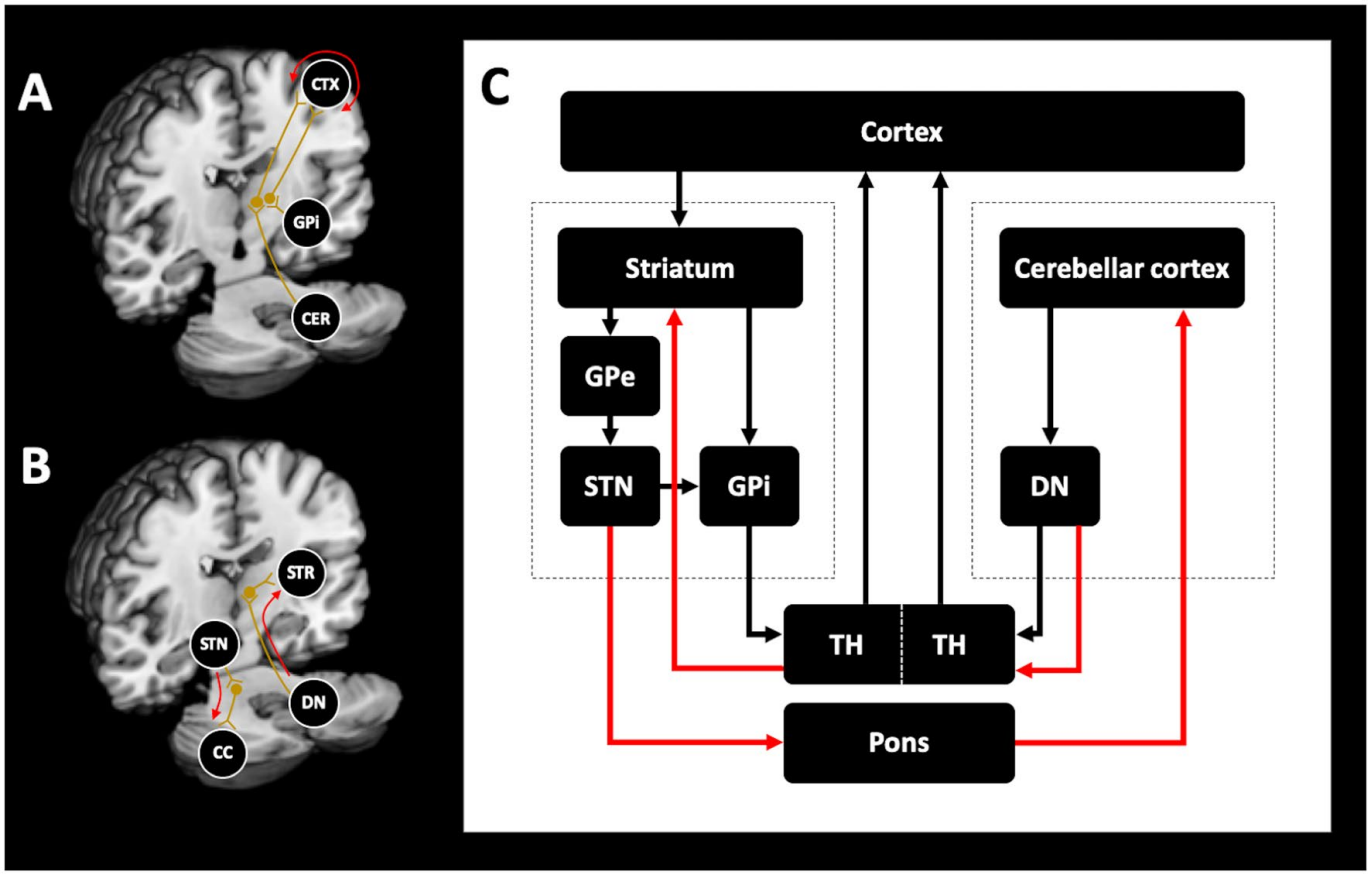
Schematic representation of brain structures involved in the pathogenesis of dystonia. Panel (**a**) shows the traditional view of distinct (striato-)pallido-thalamo-cortical and cerebello-thalamo-cortical pathways that convergently project to distinct thalamic nuclei and are only integrated at the neocortical level. Panel (**b**) shows the newly identified disynaptic anatomical connections (red arrows) linking the subthalamic nucleus with the cerebellar cortex via the pons and the dentate nucleus with the striatum via the thalamus. The contemporary model integrating the new connections (red arrows) is shown in panel (**c**). *CC* cerebellar cortex, *CER* cerebellum, *CTX* cortex, *DN* dentate nucleus,* GPe* globus pallidus externus, *GPi* globus pallidus internus, S*TN* subthalamic nucleus, *TH* thalamus

This view has been challenged by the evidence of direct anatomical connections between basal ganglia and cerebellum in animals (Bostan et al. 2010; Hoshi et al. 2005) and humans (Milardi et al. 2016) (Fig. 2). Transneuronal transport of Rabies viruses demonstrated disynaptic pathways between the dentate nucleus and the striatum (Hoshi et al. 2005), as well as the subthalamic nucleus (STN) and cerebellar cortex (Bostan et al. 2010) in brains of Macaque monkeys. The connections were shown to be dense and to affect both, motor and non-motor domains of the basal ganglia and the corresponding regions in the cerebellum. The presence of connections between STN and cerebellar cortex was confirmed in humans using diffusion tensor imaging (Milardi et al. 2016; Pelzer et al. 2013). Connections were also found between the dentate nucleus and both, the SN and pallidum, highlighting that reciprocal connections between both circuits exist and that cerebellar output may have a direct impact on basal ganglia functions and operations. Different animal models collectively confirmed an important functional relationship between basal ganglia and cerebellum (Neychev et al. 2008; Rauschenberger et al. 2020; Chen et al. 2014). The cerebellum was able to modulate striatal activity with a short latency via a disynaptic connection in mice (Chen et al. 2014). The animals developed dystonia in case of aberrant cerebello-striatal information flow. The occurrence of dystonic signs was thus dependent on the influence of both circuits which supports the hypothesis that dystonia results from a disruption of an integrated basal ganglia-cerebellar network rather than due to an isolated impairment of one of the structures (Neychev et al. 2008). This is in keeping with recent tractography studies showing differences in the anatomical connection between the pallidum and brainstem/cerebellum in cervical dystonia (Blood et al. 2012). In addition, recent fMRI studies in healthy subjects demonstrated a relationship between increased coupling of the putamen and the cerebellum with the primary motor cortex and movement speed (Pool et al. 2013) and striato-cerebellar interactions during encoding of a motor sequence task (Tzvi et al. 2015). In dystonia, functional MRI studies revealed co-existing impairments of striato-pallido-thalamo-cortical and cerebello-thalamo-cortical circuits (Filip et al. 2017; Rothkirch et al. 2018). The close interaction of the cortico-basal ganglia and the cerebello-thalamic pathway was furthermore supported by the observation that patients with essential tremor and thalamic DBS had more beneficial DBS outcome when a specific cluster within the cerebello-thalamo-cortical tract was targeted (Al-Fatly et al. 2019). Moreover, patients following thalamotomy showed an increased coupling between the frontal eye field and the cerebellum (Tuleasca et al. 2018) and pallido-cerebellar oscillatory connectivity has been linked to dystonia severity in patients who underwent pallidal deep brain stimulation (Neumann et al. 2015).

Evidence for a link between the cerebellum and the striatum on the neurotransmitter level derives from the observation that the activation of cerebellar and striatal glutamate receptors, specifically AMPA receptors, induced dystonia in animal models whereas AMPA antagonists reversed this effect and improved dystonia (Fan et al. 2012; Sander and Richter 2007). In the cerebellum, the effects were most likely exerted by the modulation of Purkinje cells which are the only efferents of the cerebellar cortex. The glutamatergic actions within the cerebellum could be related to the observed cerebellar overactivity in human and animal imaging studies although it is not clear whether the increased cerebellar outflow is disease-related or compensatory in nature. It also remains elusive whether the reciprocal connections between cerebellum and basal ganglia are affected by AMPA-mediated changes of the glutamatergic tone. The antidystonic effect of AMPA receptor antagonists is also in keeping with an increased expression of AMPA receptor subunits in reprogrammed neurons from patients with X-linked dystonia-parkinsonism who show a severe striatal atrophy (Capetian et al. 2018). Therefore, modulation of AMPA receptors could indicate an interaction of the cerebellum and basal ganglia independent of their structural connectivity.

## Conclusions and outlook

The clinical key feature of dystonia is an insufficient suppression of undesired movements either during rest or during the execution of a certain task. New evidence suggests that striosomal dysfunction could result in dysregulated dopamine release in the substantia nigra causing an imbalance between the direct and indirect pathway that is associated with impaired inhibition and the occurrence of dystonic movements. This hypothesis, however, has to be proven in future studies. Furthermore, dystonia can no longer be regarded a disorder of the basal ganglia. Recent evidence indicates that the cerebellum is likewise involved in the pathogenesis and that strong interactions between the basal ganglia and the cerebellum are present not only under physiological conditions but also in dystonia (Fig. 2). Disynaptic connections appear to be the anatomical basis for the short latency crosstalk between the cerebellum and basal ganglia. The interplay between basal ganglia and cerebellum highlights that the cerebellum is a potential treatment target for dystonia (Tewari et al. 2017). An unmet need in dystonia research is the uncertainty whether these new concepts apply to all or only a subgroup of dystonias (Tomic et al. 2020). Other poorly understood aspects are the insufficient mapping of non-motor signs and the changes in non-motor-related circuits due to dystonia.

## Data Availability

Not applicable.
